# *Pseudopus apodus* Soft Tissue Anatomy Based on Comparison of Classical Dissection and Multi-Detector Computed Tomography

**DOI:** 10.3390/ani15050615

**Published:** 2025-02-20

**Authors:** María Isabel García-Real, Encarnación Fernández-Valle, Sara Jiménez, María José Ruiz-Fernández, David Castejón-Ferrer, Andrés Montesinos-Barceló, María Ardiaca-García, Nerea Moreno, Juncal González-Soriano

**Affiliations:** 1 Department of Animal Medicine and Surgery, Faculty of Veterinary, Complutense University, Avenida Puerta de Hierro s/n, 28040 Madrid, Spain; isagreal@vet.ucm.es (M.I.G.-R.); mariru15@ucm.es (M.J.R.-F.); andmon04@ucm.es (A.M.-B.); 2 ICTS Bioimagen Complutense, Complutense University, Paseo de Juan XXIII 1, 28040 Madrid, Spain; evalle@ucm.es (E.F.-V.); dcastejon@ucm.es (D.C.-F.); 3 Achucarro Basque Center for Neuroscience, Scientific Park of the University of the Basque Country (UPV/EHU), 48940 Leioa, Spain; sara.jimenez@achucarro.org; 4 Medivet 24 Horas Los Sauces, Chamberi, Calle de Sta Engracia, 63, Chamberí, 28010 Madrid, Spain; ardiaca.m@outlook.com; 5 Department of Cell Biology, Faculty of Biological Sciences, Complutense University, Avenida José Antonio Nováis 12, 28040 Madrid, Spain; 6 Department Section of Anatomy and Embriology, Faculty of Veterinary, Complutense University, Avenida Puerta de Hierro s/n, 28040 Madrid, Spain

**Keywords:** reptiles, *Pseudopus apodus*, anatomy, computed tomography

## Abstract

Anatomy is critical for understanding the physiological and biological adaptations of living creatures. In the case of *Pseudopus apodus*, an anguimorph lizard belonging to the Order Squamata, it is particularly important considering the scarce previous works on the morphology of its coelomic cavity. It is interesting to consider that, over the years, using non-invasive approaches in reptiles, such as diagnostic imaging methods, is becoming popular for both scientific and clinical purposes. For the present work, we used a total of five *Pseudopus apodus* individuals (two males and three females); one male and one female were whole-body examined by multi-detector computed tomography (CT) and then all were dissected following a conventional anatomical protocol. The novelty and the main contribution of our multi-detector CT study is to identify structures that had never been identified before using this technique, such as the opening of the vomeronasal organ and the choanae, the tongue, the glottis, the hyoid bone, the esophagus, the stomach, the small and large intestines, the cloaca, the liver, the gallbladder, the kidneys, the ovarian follicles, the trachea, the bronchial bifurcation, the lungs, the heart, the aortic arches, the aorta, the sinus venosus, and the cranial cava veins. On the contrary, other organs like the thyroid, the pancreas, the spleen, the ureters, the urinary bladder, the oviducts, the testes, the hemipenes, the pulmonary trunk, and the pulmonary arteries were only identified in the anatomical dissection. Thus, our results demonstrate that multi-detector CT scanning is a useful tool to identify a significant number of anatomical structures in *Pseudopus apodus*, which is important for clinical veterinary practice related to this species or different conservation programs, among other applications.

## Simple Summary

The class Reptilia, is a group of air-breathing vertebrates that have internal fertilization, amniotic development, and epidermal scales covering part or all of their body. The major groups of living reptiles—turtles (order Testudines), tuatara (order Rhynchocephalia), lizards and snakes (order Squamata), and crocodiles (order Crocodylia, or Crocodilia)—account for over 10,000 species. The species object of our interest is the lizard *Pseudopus apodus*, a large, legless lizard, most active at night, which is broadly distributed but rarely seen. Here, we describe the anatomy of the coelomic cavity of this animal by combining traditional dissection and a multi-detector CT scanning. Our study demonstrates that, by using this non-invasive method, it is possible to identify most of the anatomical structures which are found in the celomic cavity of this species. In consequence, the main contribution of this work is to carry out the first anatomical description of the coelomic cavity of *Pseudopus apodus* by means of an imaging technique.

## 1. Introduction

Reptiles are one of the most interesting groups among vertebrates in terms of their diverse morphology and characteristics. There are more than 10,000 living species, classified into four orders: Crocodilia (crocodiles and alligators), Sphenodontia or tuataras, Squamata (lizards and snakes), and Testudines (turtles and tortoises) [1]. Within the Squamata group, there is the anguimorph lizard of our interest: *Pseudopus apodus* or the European glass lizard [2], which is the biggest representative of the Anguidae family in terms of its distribution range.

These individuals can reach a length of 120 cm, including a very long tail. Although often referred to as a legless lizard, it is occasionally possible to distinguish two small rear legs, visible near the cloaca, that measure around 2 mm or even less. The vulgar Italian name “Lucertola di vetro” (=glass lizard), instead, refers to the characteristic of the loss of the tail, which divides into many small pieces when is autotomized [3,4].

It must be taken into consideration that the use of non-invasive approaches for the study of anatomy, such as diagnostic imaging techniques, is becoming very popular, especially for physiological and pathological purposes. There are previous publications related to the use of micro-computed tomography systems concerning the axial and appendicular skeleton of *Pseudopus apodus* [5,6,7,8,9], with part of them being from fossil specimens [10,11,12,13]. However, there are no previous references on multi-detector computed tomography (CT) usage to describe the coelomic cavity of this lizard. For this reason, we conducted a study on the lizard *Pseudopus apodus*, comparing the traditional anatomical dissection and the use of the multi-detector CT technique. Our objective was to determine if this diagnostic technique, which is otherwise widely used in clinical veterinary practice, could be a useful tool to analyze the coelomic cavity of this legless reptile.

## 2. Material and Methods

**Figure 1:**
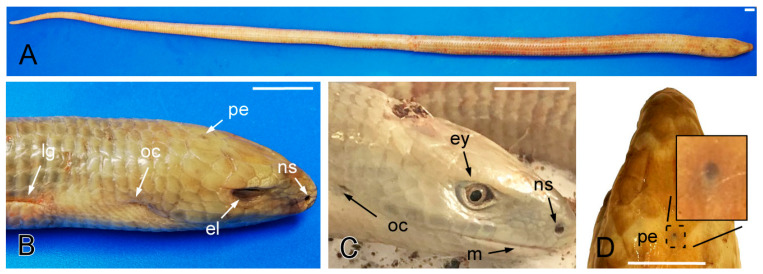
The dorsal (**A**,**B**,**D**) and lateral (**C**) images of the *Pseudopus apodus* specimen. The lateral groove, mouth, nostrils, eyes, eyelids, otic clefts, and parietal eye are identified. See the list for abbreviations. Bar = 10 mm.

For the present study, we used 5 healthy young adult individuals, between 5 and 10 years of age (2 males and 3 females), from *Pseudopus apodus* (with good overall appearance, shiny skin without lacerations, damage, or changes in the color, as well as normal motility and standard reflexes) (Figure 1). All the lizards were obtained from commercial pet suppliers. Animals were handled in accordance with the guidelines for animal research set out in the European Community Directive 2010/63/EU [14] and following the recommendations of the European Commission for the protection of animals used for scientific purposes. All procedures were approved by the local ethics committee (O.H. (CEA) UCM—NP0409032022-2022). Whole-body CT scans were performed on 2 animals (1 male and 1 female). Sedation was induced with isoflurane via chamber induction. The animals were placed in a zip bag filled with oxygen mixed with 5% isoflurane. Induction was considered successful when the righting reflex was lost. This point was reached in approximately 5 min. The animals were then removed from the bag, placed on the table fully extended in sternal recumbency, and secured with paper tape. Sedation was maintained with 3% isoflurane in pure oxygen, with the snout placed near the end of the Ayres T-piece of the anesthetic circuit. At the end of the procedure, the isoflurane was discontinued and both animals recovered uneventfully within 10 min. A Toshiba Aquilion 64 Multislice CT Scanner (200 mA, 120 kV; 512 × 512 matrix and a 10.2 cm field-of-view diameter) was used. High-resolution bone and soft tissue reconstruction algorithms were applied with the following parameters: WL 550, WW 2550, slice thickness 0.5 mm, and a reconstruction interval of 0.4 mm (bone); WL 50, WW 500, slice thickness of 0.5 and 1 mm, and a reconstruction interval of 0.4 and 0.8 mm (soft tissue). Additionally, a post-acquisition lung reconstruction algorithm was used. Acquisition with the soft tissue algorithm was repeated 60 and 900 s after the IV contrast injection. Non-ionic iodine contrast medium (Optiray 300 mg/mL; Guerbet Ireland) was used. The contrast was manually injected at a dose of 600 mg I/kg into the ventral coccygeal vein using a 25G butterfly needle with an extension set, followed by a 1 mL saline bolus. Partial extravasation of the contrast was observed in one animal.

All images were analyzed using the Radiant DICOM Viewer (Medixant. Radiant DICOM Viewer, Version 2021.1. https://www.radiantviewer.com, accessed on 27 June 2021) software by two veterinarians with ample experience in reptile diagnostic imaging. They were initially evaluated in the transverse plane and later reconstructed by postprocessing in dorsal and sagittal planes using multiplanar reconstruction (MPR); 3D volume rendering reconstructions were also performed.

The anatomical study was carried out in the 5 individuals using a conventional dissection technique. For this purpose, sedated animals were sacrificed with an overdose of anesthesia, and after an incision was made in the midline of each animal, placed in supine decubitus. Then, a first analysis of the superficial plane of the coelomic cavity was conducted, for subsequent access to a deeper plane to study each of the structures of interest. In all cases, their morphology, disposition, and relations were considered.

## 3. Results

Regarding its morphology, the *Pseudopus apodus* is dark colored, paler on the ventral surface and the head, with a ring-like/segmented appearance that makes it look like a big earthworm (Figure 1A). All individuals show a distinctive fold of skin down each side, known as the lateral groove. They all have a narrow head, with a rounded muzzle in the dorsal view and an elongated muzzle in the lateral view, and large eyes with rounded pupils and eyelids. All *Pseudopus apodus* individuals have unmovable lips, composed of hard skin, with a pair of nostrils or nares, located dorsally (Figure 1B). They communicate with the nasal cavity (beginning of the upper airways). Laterally, it is possible to observe the otic cleft, which indicates the location of the ear (Figure 1B,C), while dorsally it is possible to see the parietal eye or third eye, which is typical in many individuals of the Anguidae family (Figure 1D).

**Figure 2:**
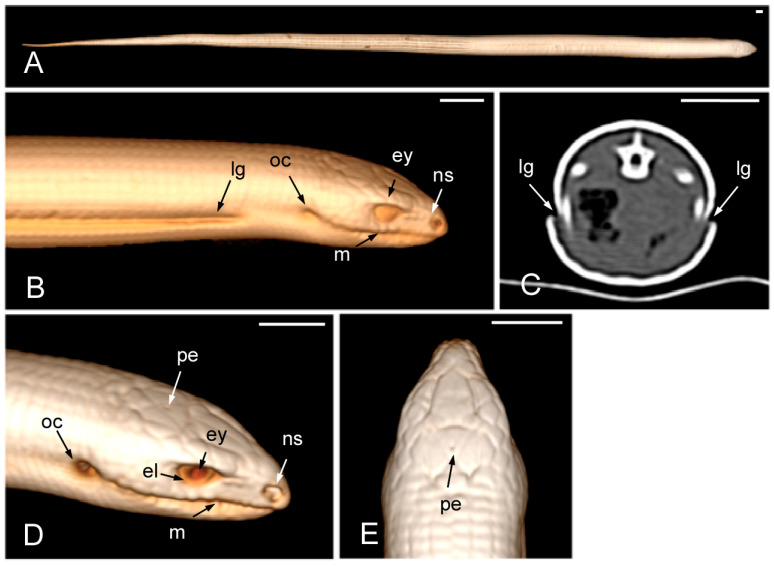
The 3D volume rendering (**A**,**E** in the dorsal view, **B**,**D** in the lateral view) and transverse 2D (**C**) CT images of a specimen of *Pseudopus apodus*. The lateral groove, mouth, nostrils, eyes, eyelids, otic clefts, and parietal eye are identified. See the list for abbreviations. Bar = 10 mm.

The 3D volume rendering CT images provide a detailed view of the surface anatomy of these individuals (Figure 2A). The lateral groove was readily identifiable in the 3D images (Figure 2B) as well as in the 2D transverse plane images (Figure 2C), in which it was even more evident. The hyperattenuating appearance of the osteoderms layer suggests a high content of bone in them (Figure 2C). The mouth, the nostrils, the eyes, the eyelids, and the otic clefts were easily identified in the lateral view of the 3D volume rendering images (Figure 2B,D) while the parietal eye was also observed in the dorsal view (Figure 2E).

**Figure 3:**
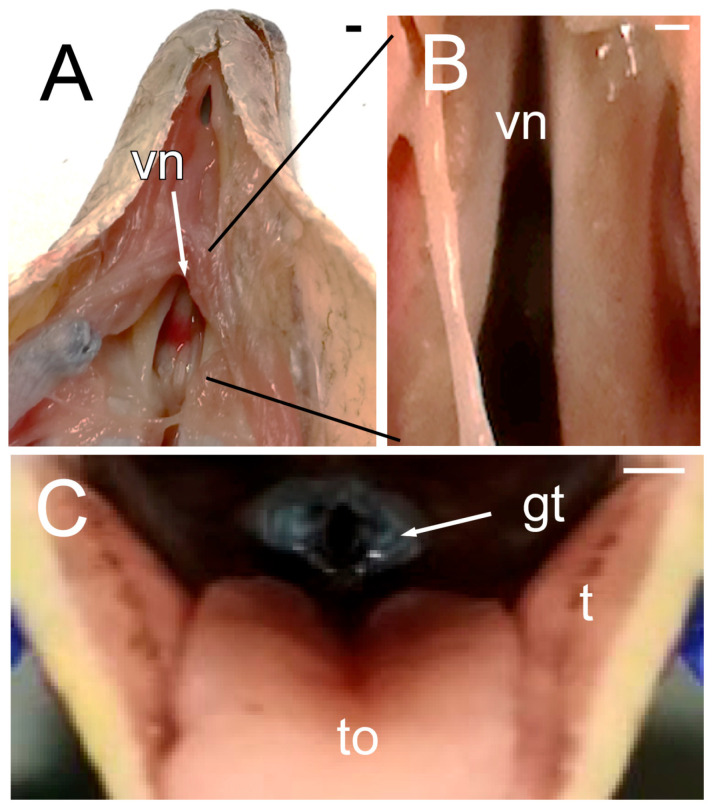
The images of the oral cavity of *Pseudopus apodus* showing the opening of the vomeronasal organ and the choanae (**A**,**B**), the tongue (**C**), and the glottis (**C**). See the list for abbreviations. Bar = 1 mm.

In the oral cavity, a wide overture that coincides with the vomeronasal organ and the choanae opening stands out (Figure 3A,B). The presence of the glottis, quite cranial in the oral cavity, and visible when the mouth is opened (Figure 3C), is noteworthy. The upper airways also include a larynx and pharynx that do not show special details. To complete the macroscopic description of the oral cavity, the cone-shaped, slightly asymmetrical teeth and the forked tongue should also be highlighted (Figure 3C).

**Figure 4:**
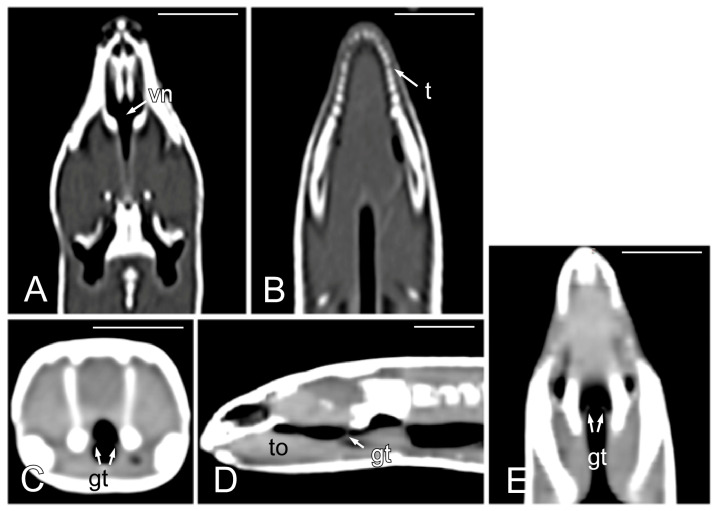
The CT images of *Pseudopus apodus* in the dorsal (**A**,**B**,**E**), transverse (**C**) and sagittal (**D**) planes showing the opening of the vomeronasal organ and the choanae (**A**), teeth (**B**), tongue (**D**) and glottis (**C**–**E**). See the list for abbreviations. Bar = 10 mm.

The opening of the vomeronasal organ together with the choanae was identified in the dorsal plane as a V-shaped structure delimited by thin hyperattenuating margins (Figure 4A). The teeth were more distinctly evaluated in the dorsal plane (Figure 4B). The tongue appeared as a hypoattenuating structure located in the ventral part of oral cavity and was more clearly visible in the sagittal plane (Figure 4D). The glottis (Figure 4C–E) was identified as a thin, soft tissue structure with a central opening between the oral and pharyngeal cavities. Although it was visible in the transverse, sagittal, and dorsal planes, it was easier to identify in the latter two.

**Figure 5:**
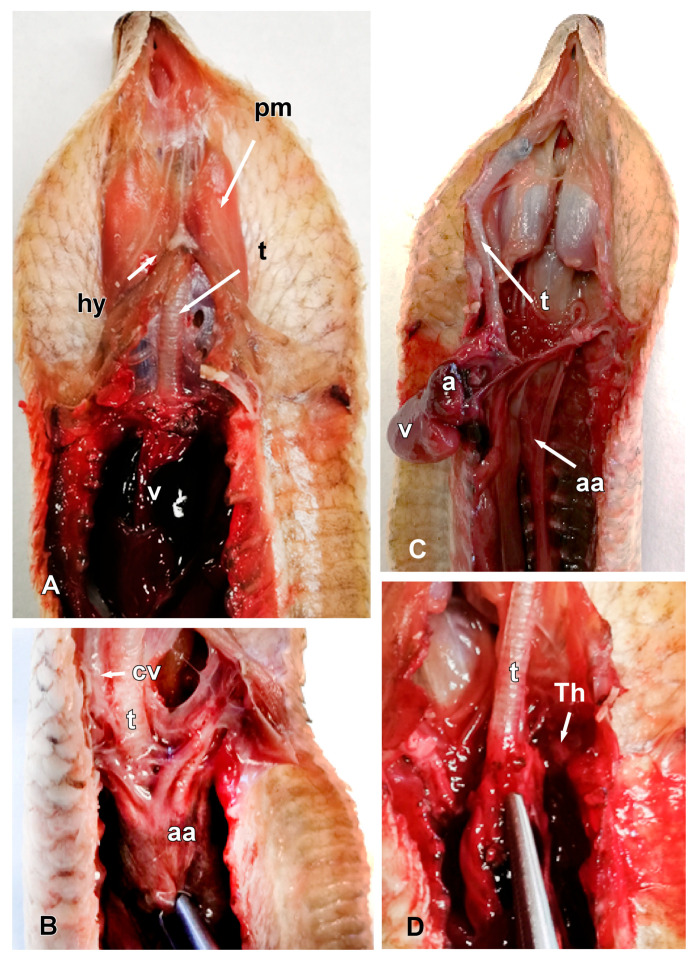
The images of the dissection of *Pseudopus apodus* in the ventral plane showing the hyoid bone (**A**), the trachea (**A**–**D**), the cranial cava vein, and aortic arches (**B**), the atria and ventricle (**A**,**B**), and the thyroid gland (**D**). See the list for abbreviations. Bar = 10 mm.

In general terms, the internal organs in many serpentiform species are elongated, arranged sequentially, or even compressed laterally. This is also true in the case of the *Pseudopus apodus*. Once the animal is opened, along the ventral midline, the presence of two powerful masticatory muscles (pterygoideus muscles) (Figure 5A,B), together with the hyoid bone that provides support to the tongue (Figure 5A), is notable. Only a portion of the hyoid bone can be seen in a dissection, compared to the full shape of the in situ bone with CT imaging (see below). The trachea is a membranous and cartilaginous flexible duct that extends from the larynx to the entry of the thoracic cavity (Figure 5A–D). As in other lizards, the tracheal rings are incomplete. Parallel to this, it is possible to identify the presence of two cranial cava veins. The heart is elongated and conically shaped, with two atria and a single ventricle (Figure 5B). There are two aortic arches (left and right), emerging from the heart, and a pulmonary trunk, which is subsequently divided into two pulmonary arteries (right and left). The aortic arches join to form the dorsal aorta, which runs through the entire coelomic cavity (Figure 5B,C). Immediately above the heart, it is possible to see the thyroid gland, which is small and rounded (Figure 5D).

**Figure 6:**
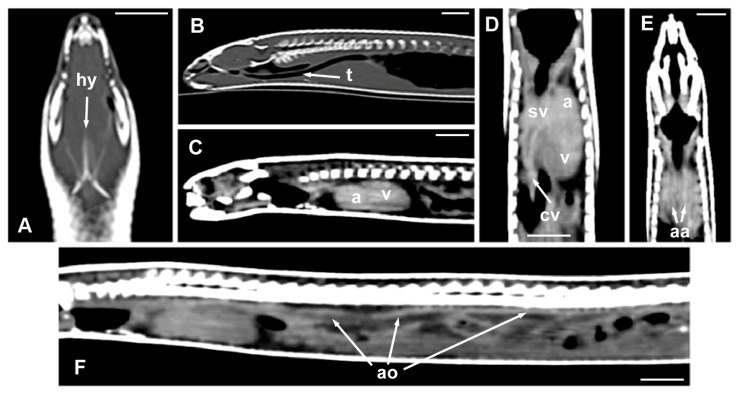
The CT images of *Pseudopus apodus* in the dorsal (**A**,**D**,**E**) and sagittal (**B**,**C**,**F**) planes showing the hyoid bone (**A**), the trachea (**B**), the atria and ventricle (**C**,**D**), the sinus venosus and the two cranial cava veins (**D**), the aortic arches (**E**), and the dorsal aorta. The images (**C**–**F**) were obtained after contrast media administration. See the list for abbreviations. Bar = 10 mm.

The morphology of the hyoid bone was most clearly observed in the dorsal plane, in the CT images obtained from the MPR reconstruction (Figure 6A). The trachea was clearly distinguished as a tubular structure containing gas in the lumen, extending from the glottis to the main bronchial bifurcation; the sagittal plane allowed the visualization of its entire length (Figure 6B). The heart and some great vessels were best identified in postcontrast images (Figure 6C–F). The dorsal and sagittal planes were the most suitable for observing the slight distinction between the atria and the ventricle of the heart (Figure 6C,D). On the contrary, the sinus venosus, the two cranial cava veins, and the aortic arches were most clearly observed in the dorsal plane (Figure 6D,E). The aorta was identified along the midline, just ventral to the spine, and was best observed in the sagittal plane (Figure 6F). The pulmonary trunk, divided into the left and the right pulmonary arteries, and the thyroid gland, were not identified in the CT images.

**Figure 7:**
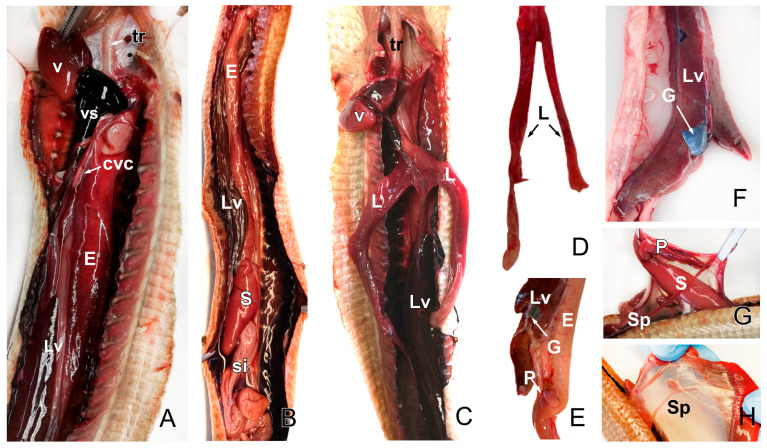
The images of the dissection of *Pseudopus apodus* showing the components of the digestive system, including the esophagus (**A**), the liver, the stomach, the small intestine, the pancreas, and the spleen (**B**–**H**). The images also show parts of the respiratory system, including the trachea and the lungs (**A**–**C**). Image (**D**) shows a dissection of the lungs and the gallbladder (**E**,**F**). See the list for abbreviations. Bar = 10 mm.

Venous blood from the posterior part of the coelomic cavity returns to the heart via a caudal cava vein, which together with the cranial cava veins, flows into the venous sinus (widening of the right atrium) (Figure 7A). The trachea extends from the larynx to the entrance of the thoracic cavity. It divides into two primary bronchi (left and right) that enter the lungs. The sac-like lungs are paired and elongated (Figure 7C) and are situated dorsocaudally to the heart and dorsally to the cranial parts of the liver and stomach. When comparing the left and right sides, there is a clear asymmetry with respect to their lengths: the left lung was shorter and reduced compared to the right (Figure 7D). The liver is an elongated structure, topographically related to the esophagus, stomach, and lungs (Figure 7A–C). Towards the middle of its course, it is possible to observe the gallbladder (Figure 7F). With an elongated morphology, the esophagus flows into the stomach (Figure 7G), which continues with the small intestine (Figure 7B). The spleen is, in relation to the stomach, the small intestine (between the intestinal serosa) (Figure 7G,H), and the pancreas, very visible at the beginning of the small intestine (Figure 7G).

**Figure 8:**
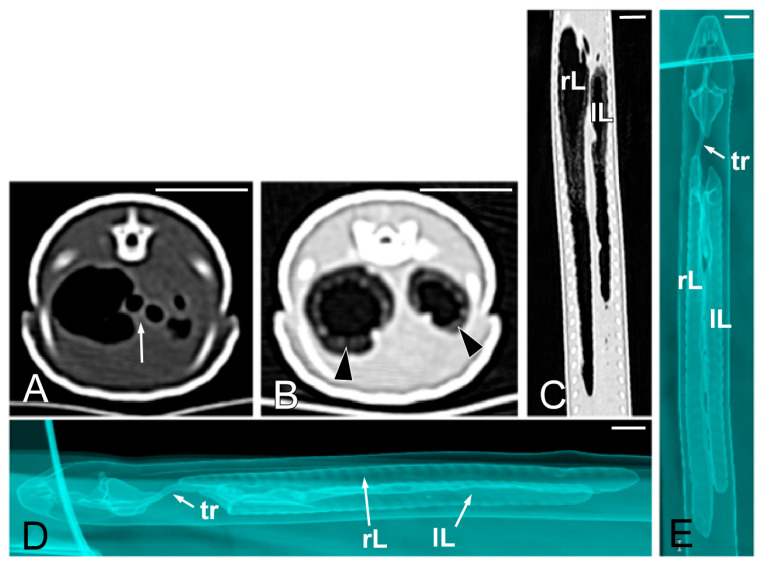
The CT images of *Pseudopus apodus* in the transverse (**A**,**B**) and dorsal (**C**) planes, and the 3D volume rendering showing the airways (**D**,**E**). Image (**A**) was obtained with a soft tissue algorithm and shows the main bronchi bifurcation (white arrow), while images (**B**,**C**) were postprocessed using a lung algorithm and show the peripheric hypoattenuating area (black arrowheads) around an air-filled cavity. The images (**D**,**E**) were postprocessed using the 3D volume rendering with airways algorithm and offer a detailed representation of the trachea (tr) and lungs (L). Bar = 10 mm.

**Figure 9:**
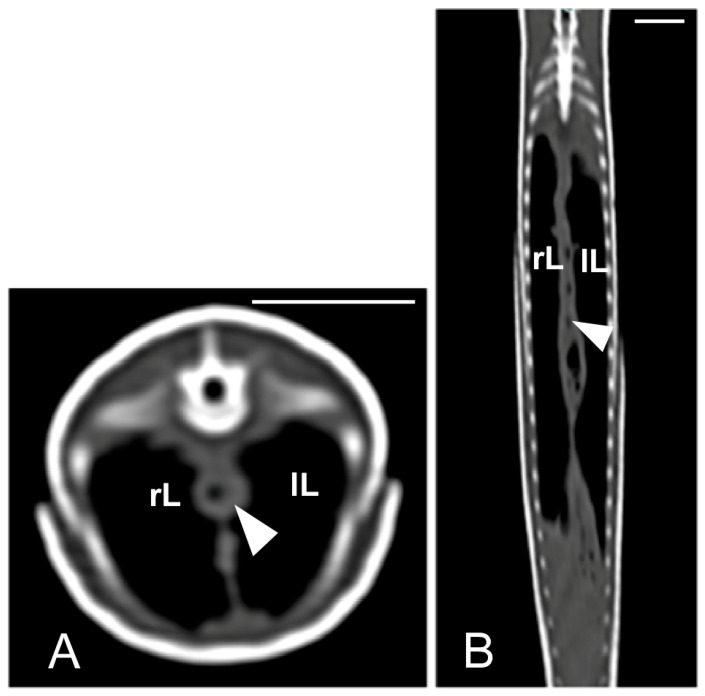
The CT images of *Pseudopus apodus* in the transverse (**A**) and dorsal (**B**) planes showing segments of the esophagus (arrowheads) with intraluminal gas. The esophagus is located between the lungs (L). Bar = 10 mm.

**Figure 10:**
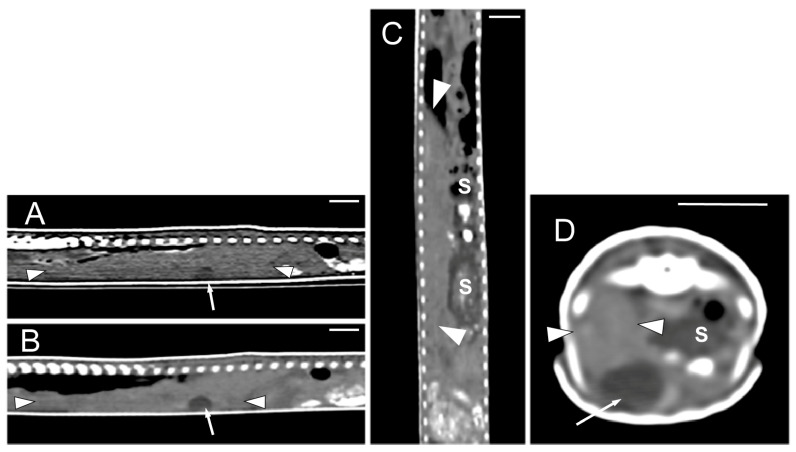
The CT images of *Pseudopus apodus* in the sagittal (**A**,**B**), dorsal (**C**) and transverse (**D**) planes showing the liver (between arrowheads) and gallbladder (arrow). Image (**A**) was obtained precontrast administration and images (**B**–**D**) were obtained postcontrast. The contrast enhancement of the normal hepatic parenchyma improved the differentiation between it and the gall bladder and between the liver and stomach (S). Bar = 10 mm.

**Figure 11:**
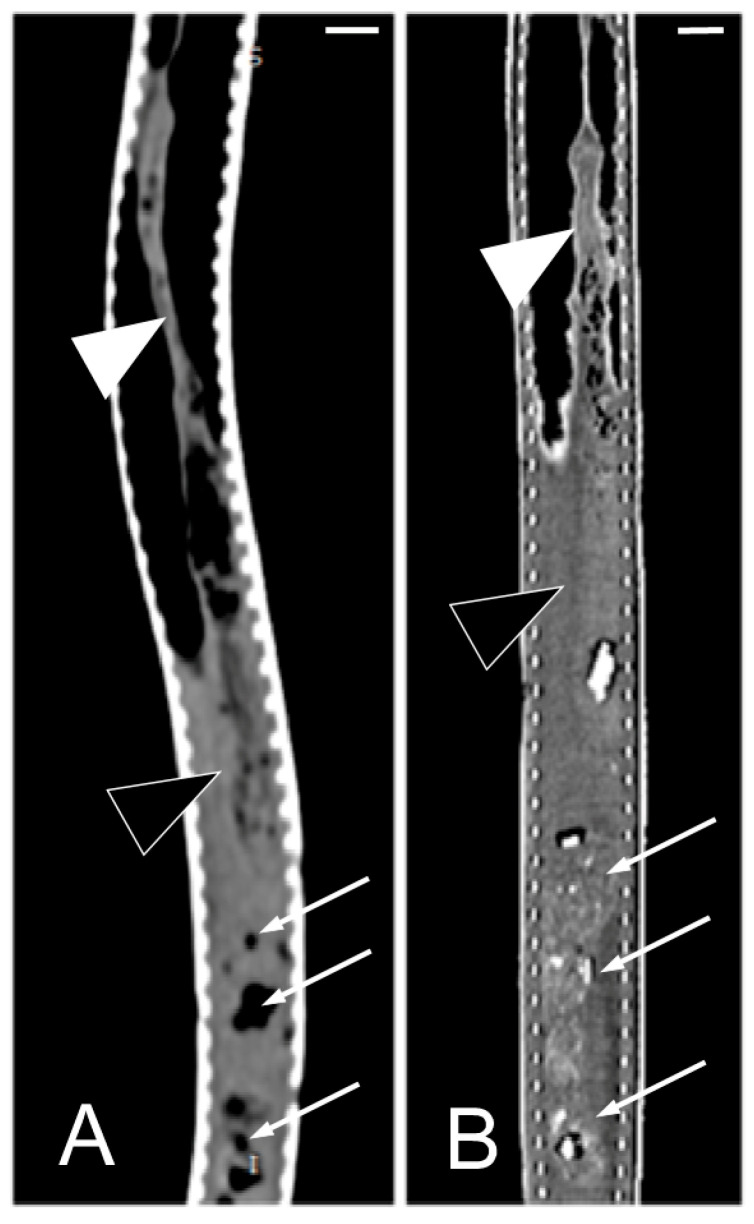
The CT images of *Pseudopus apodus* in the dorsal plane showing segments of the esophagus (white arrowheads), stomach (black arrowheads), and intestine (white arrows) with intraluminal gas (**A**) and with gas and hyperattenuating content (**B**). Bar = 10 mm.

Concerning the CT images, the bifurcation of the trachea into the main bronchi was most clearly observed in the transverse plane (Figure 8A). The two lungs appeared as elongated structures with a reticular hypoattenuating area around an air-filled cavity. The distinction between the hypoattenuating area and just air was more distinctly identified in the transverse and dorsal planes using a postprocessing lung algorithm (Figure 8B,C). The 3D volume rendering using a postprocessing airways algorithm detailed the trachea and lungs (Figure 8D,E). Between the lungs, it was possible to identify those esophageal segments containing gas. On the contrary, those esophageal segments without intraluminal gas were indistinguishable from the surrounding soft tissues. The transverse and dorsal planes were the most useful for recognizing the esophagus (Figure 9). The liver appeared as an elongated organ with soft tissue attenuation located ventral to the caudal midsection of the lungs, on the right side of the stomach. Its margins were not well defined. The gall bladder was identified in the ventral part of the coelomic cavity as a hypoattenuating small oval structure surrounded by the hepatic parenchyma (Figure 10A). The differentiation between the gall bladder and the hepatic parenchyma was more evident in the postcontrast studies (Figure 10B–D). As in the case of the esophagus, the stomach and the small and large intestines were easily distinguished when containing intraluminal gas (Figure 11A) or hyperattenuating content (residual ingesta) (Figure 11B). The small and large intestines were located just caudal to the liver and the stomach, occupying approximately the caudal third of the coelomic cavity. The spleen and pancreas could not be identified when using CT.

**Figure 12:**
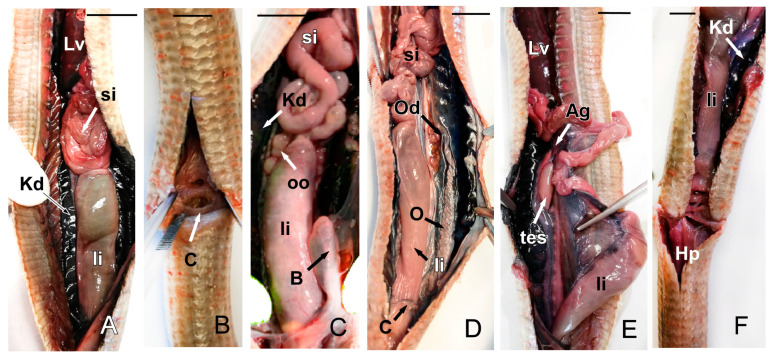
The images of the dissection of *Pseudopus apodus* showing the kidney, the cloaca, the bladder (**A**–**C**), and the ovarian system (**C**,**D**), including the oocites, an ovary duct, and an ovary (**B**–**E**). In the case of a male, it is also possible to see the testicles and the hemipenes (**E**,**F**). See the list for abbreviations. Bar = 10 mm.

The small intestine continues with the large intestine, which flows into the cloaca with the urinary and genital tracts (Figure 12A). The kidneys are elongated and multilobed. One may be more advanced than the other or at the same level within the coelomic cavity (Figure 12A,C,F). A pair of ureters emerge from them, which lead to the cloaca, practically to the edge of the bladder. Macroscopically, the bladder resembles a fold of mucosa rather than a urinary bladder per se (Figure 12C). The gonads were identified in the dorsal part of the cavity, cranial to the kidneys in both males and females. The female reproductive system mainly consists of two ovaries and oviducts. The pair of ovaries showed numerous follicles, visible on the ovary surface. The oviducts were observed as two tubes, relatively long and coiled, that flow into the cloaca separately (Figure 12C,D). The male reproductive apparatus consists of a pair of testes, identified as two ovoid-elongated shaped structures, efferent ducts, and two urinogenital ducts that open into two hemipenes that are sac-like and lack erectile tissue. They are inversely stored in the base of the tail (Figure 12E,F) and may produce bulges in the ventral proximal tail.

**Figure 13:**
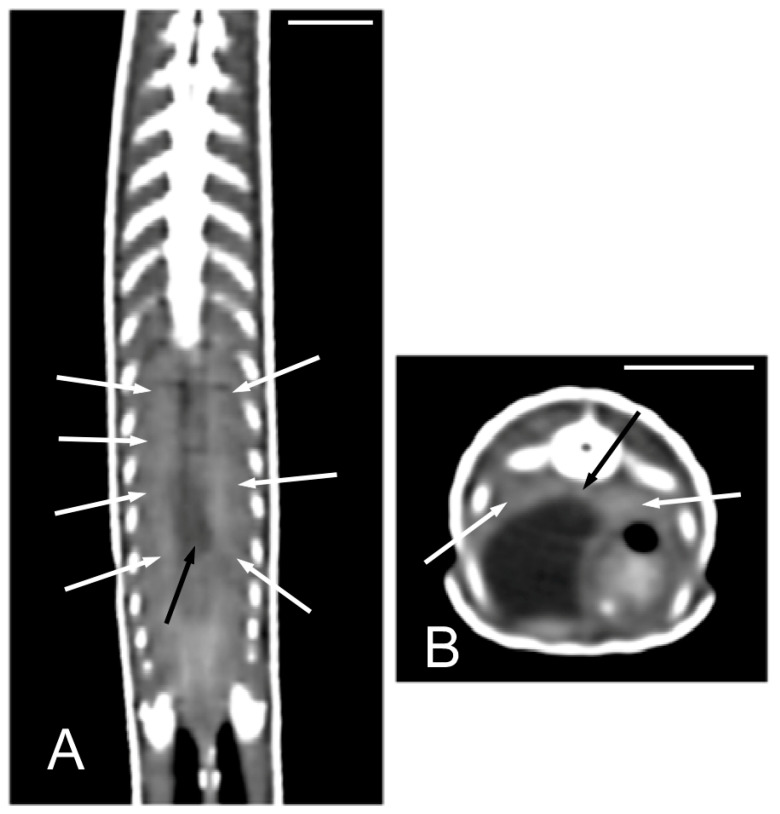
The postcontrast CT images of *Pseudopus apodus* in the dorsal (**A**) and transverse (**B**) planes showing both kidneys (white arrows) and the aorta (black arrow) between them in the dorsal part of the coelomic cavity, just ventral to the spine. Bar = 10 mm.

**Figure 14:**
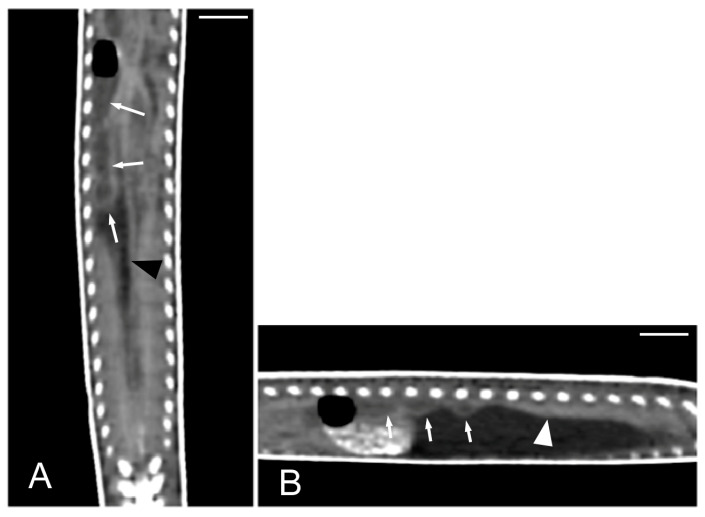
The postcontrast CT images of *Pseudopus apodus* in the dorsal (**A**) and sagittal (**B**) planes showing ovarian follicles (arrows), just cranial to the kidney. In image A, the aorta (black arrowhead) is identified in the midline. In image B, the white arrowhead points to the right kidney. Bar = 10 mm.

The kidneys and ovaries were identified only in postcontrast CT images. The kidneys appeared as a pair of soft tissue attenuating elongated structures in a ventrolateral position to the spine, in the caudal third of the coelomic cavity. They were best seen in the transverse and dorsal planes. Their margins were not well defined. The aorta was only seen in the midline between them (Figure 13). We could distinguish ovarian follicles only in one individual and they appeared as hypoattenuating rounded to oval structures, bounded by fine slightly hyperattenuating margins (Figure 14). The ureters, urinary bladder, oviducts, testes, and hemipenes were not recognizable in our CT studies.

**Figure 15:**
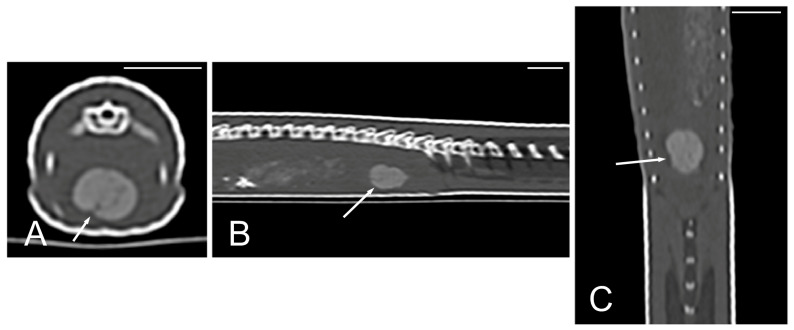
The CT images of *Pseudopus apodus* in the transverse (**A**), sagittal (**B**), and dorsal (**C**) planes showing hyperattenuating content (urate salts) (arrows) in the cloaca. Bar = 10 mm.

The cloaca was identified in the caudoventral part of the coelom in only two animals due to the presence of hyperattenuating content in the lumen (urate salts) (Figure 15).

## 4. Discussion

### 4.1. Anatomy

In our study, postcontrast imagery offered a better differentiation of the liver, kidneys, heart, and great vessels. The anatomy of *Pseudopus apodus* was first described by Pallas [2]. Since then, data concerning the anatomy is scarce, particularly regarding the coelomic cavity of this animal—hence the importance of the present work. Thus, the aim of this paper is worthwhile, pointing out some of the anatomical features of these animals in comparison with other squamates. Regarding external morphology, one of the most important details concerns the possible sexual dimorphism. In the case of the animals used in the present work, identifying males from females using their external aspects was rather challenging. Our observations coincide with what has already been stated by Kukushkin and Dovgal [15], who indicated there are usually subjective external details, such as the bulging and externalization of the hemipenes, or the reaction when the animals are handled. However, they state that, to make an accurate classification separating males and females, it is essential to carry out allometric studies.

In relation to some of the internal organs, the basic pulmonary morphology in the *Pseudopus apodus* agrees with what has been described by earlier authors for this species [2,16,17]. According to our results, the sac-like lungs are paired and elongated, with an asymmetry between the length of the right and the left side when both sides are compared. The first formal description of *Pseudopus apodus* by Pallas [2] already provided information on pulmonary asymmetry for this species. According to our results, this author indicates that the right lung is longer and more extended than the left, contrary to the results described by Lambertz [18]. These authors found specimens in which the right lung was shorter and more reduced than the left one, and also the reverse, apparently without any specific pattern. Interestingly, most snakes also exhibit a significant reduction in their left lung, and quite a few lack it entirely [19]. Also, amphisbaenians frequently exhibit a reduction in their right lung [20,21,22,23,24], which could indicate that both alternatives are suitable adaptations to a serpentiform habitus.

The heart, together with the digestive and reproductive systems of *Pseudopus apodus* follow a similar pattern to that described for other reptiles of the same Order squamata [25].

### 4.2. Imaging Diagnosis

Multi-detector CT is one of the most important diagnostic imaging techniques used in human and veterinary medicine. Nowadays, it is routinely used in exotic pets as well [26]. Gumpen-Berger and Henninger reported already in 2001 that CT is an ideal technique to diagnose skeletal diseases and soft tissue enlargements in avian and reptile populations [27]. CT has been used to describe the normal anatomy of several reptiles [28,29,30,31,32,33,34,35,36,37,38,39,40,41,42,43]. This information may be used as a baseline for the clinical evaluation of unhealthy animals [30,44]. CT scans provide a clear visualization of bone structures. However, the lack of diffuse fat around the visceral organs in reptiles reduces soft tissue contrast, making their differentiation more challenging [29,44]. This factor is even more relevant in species with a narrow axial diameter, like *Pseudopus apodus*.

Almost half of the published studies on reptile CT anatomy include a correlation with cross-section anatomy [28,29,34,41,42] or both anatomic dissection and cross-section of specimens [32,33]. In our case, we choose the dissection technique since no previous bibliographic references were found for this basic type of anatomic description of *Pseudopus apodus*.

Some authors recommend scanning lizards with the long axis of the animal positioned perpendicular to the longitudinal axis of the CT table. In other words, these authors suggest scanning the individuals in latero-lateral direction instead of the standard cranio-caudal direction [34,45,46]. In this way, it is possible to obtain images of the whole anatomy of the animals with shorter acquisition times and less radiation exposure for the patient [27]. This was not possible in the case of *Pseudopus apodus* because of its length. In general, there are previous data on 1–2 mm slice thickness for reptile CT studies [27,29,30,31,32,33,34,36,37,38,39,41,43,45], or even 0.75 mm [46], 0.625 mm [42], or 0.5 mm [40]. Concerning the scanners, most of the previous reptile multi-detector CT studies refer to the use of 16-slice helical scanners [29,35,37,38,39,41,42,43,46]. In the present work, we have used a 64-slice helical CT. The consequences are shorter scan times and the possibility of selecting a lesser slice thickness (0.5 mm). In other words, the lower the slice thickness, the higher the definition of the MPR and 3D volume rendering images. The reduction in the scan times with 64-slice CT is especially important in exotic pets to minimize the time of anesthesia.

The use of intravenous iodinated contrast media can enhance certain tissues and improve the differentiation of large vascular structures in exotics [44]. Although some authors recommend only obtaining postcontrast images in reptiles to reduce the scan time [34], we performed pre and postcontrast examinations. In our study, postcontrast imagery offered a better differentiation of the liver, kidneys, heart, and great vessels. Other authors have reported improvements in the visualization of the coelomic parenchymal organs of green iguanas, black and white tegus, bearded dragons [34], red-eared terrapins [37], and chameleons [42] after intravenous contrast administration.

The slow respiratory rate, together with enhanced contrast between lung parenchyma and air, has been reported as a factor to obtaining exceptional CT images of chelonian and snake respiratory tracts [29,30,31,44]. In our work, the trachea, the main bronchi bifurcation, and the lungs could be perfectly differentiated in 2D and 3D volume rendering images. The sagittal plane was the most adequate for evaluating the entire trachea, the transverse plane for identification of the main bronchi bifurcation, and the transverse and dorsal planes to examine the lungs in 2D images. Ricciardi et al. [38] suggest that CT may be a potentially suitable technique for diagnosing a great variety of pulmonary pathologies in reptiles, whereas Gumpenberger [46] considers CT as the gold standard for images of the respiratory tract in all reptiles. This is because it allows for precise visualization of the upper respiratory tract, but especially of the variable delicate architecture of the lungs in the different species, especially with lung or bone algorithms. In our work, only the lung algorithm allowed us to differentiate the peripheric reticular hypoattenuating area from the air-filled central cavity in the lungs. The trachea and main bronchi bifurcation were easily identified in our study due to the air content.

Although we could identify the liver, the margins of the organ could not be clearly distinguished in precontrast images. The gall bladder was differentiated as a small hypoattenuating structure surrounded by hepatic parenchyma. The difference in attenuation between them was more evident in postcontrast images. It has been reported that the liver lobes and the gallbladder can be distinguished in normal loggerhead sea turtle CT images, although the distinction between the hepatic parenchyma and pectoral musculature may not be clear due to lack of contrast [29]. In a study performed with green iguanas, black and white tegus, and bearded dragons the liver was clearly distinguishable from the surrounding fat bodies. The gallbladder was identified in the three species as well [34]. Nardini et al. [35] evaluated the liver parenchyma and perfusion using dynamic contrast-enhanced computed tomography in captive green iguanas. They reported that unenhanced CT scans provided an excellent visualization of the lizard’s liver, while contrast-enhanced CT permitted further investigation of hepatic perfusion. Sochorcová et al. [37] also studied the feasibility of contrast-enhanced CT for the liver, the gallbladder, and the urogenital tract in red-eared terrapins. These authors determined that the maximum contrast enhancement of the liver was detected 60 s after the contrast injection. In our study, we saw the liver located ventral to the lung, on the right side in relation to the stomach and cranial to the intestines. Melero et al. [42] used the same anatomic landmarks to delineate the hepatic parenchyma as well as the cardiac apex in chameleons, which is just cranial to the liver in this reptile. In some reptiles, contrast-enhanced CT allows to differentiate a thin gallbladder wall [37,42], which we could not distinguish in our study.

Concerning the reptile digestive tract CT evaluation, some authors recommend the oral administration of contrast medium, as the gas-filled stomach is easy to see, yet the intestines are difficult to differentiate [27]. In our study, we performed the CT studies without oral contrast administration. We clearly identified those segments of the esophagus and intestine that had intraluminal gas or hyperattenuating content. However, there were other segments that showed a similar attenuation in comparison with other surrounding coelomic soft tissues. This fact made differentiating these esophageal and intestinal segments much more difficult. Instead, the stomach was easier to identify using the liver as a landmark, especially in postcontrast images. Valente et al. [29] reported the difficulty of distinguishing abdominal soft tissue structures, such as intestine portions, because of their small size and/or the lack of contrast between them and the surrounding tissues. In a study performed to evaluate the CT features of the coelomic cavity in green iguanas, black and white tegus, and bearded dragons [34], the stomach was well distinguished in the three species; individual small intestine loops were evident only in the green iguana, while the large intestine was particularly obvious in black and white tegus due to hyperattenuating content. In the study by Melero et al. [42], performed in chameleons, the esophagus and stomach were easy to identify, and intestinal loops were seen as tubular structures with a soft tissue attenuation wall and intraluminal small gas bubbles; however, in the same study the different intestinal segments could not be identified and only the colon could be distinguished with gas or feces when it was distended.

In our study, we identified the cloaca in the caudoventral coelom by the presence of hyperattenuating urate salts accumulated in its lumen, which coincides with the description given by Melero et al. [42] in chameleons. In the study by Banzato et al. [34] the cloaca was identified in green iguanas and black and white tegus when it was markedly distended with fluid content.

In our case, only the kidneys were identified between the urinary organs. They were best seen in postcontrast images in transverse and dorsal planes, using the spine as an anatomic landmark. Being paired organs makes their identification easier. In the study by Banzato et al. [34], who obtained only postcontrast CT images, the renal parenchyma showed an in-homogeneous appearance in the green iguanas and common black and white tegus, while it appeared quite homogeneous in bearded dragons. Sochorcová et al. [37] reported that in contrast-enhanced CT of red-eared terrapins, the maximum contrast enhancement of the kidneys was detected 20 s after the contrast injection; the kidneys showed a homogeneous contrast enhancement, and the ureters could be clearly identified in postcontrast images of this reptile. In the CT study performed by Melero et al. [42], carried out in chameleons, the kidneys appeared as lobulated, oval, bilateral and symmetric structures, with soft tissue attenuation. They were more easily identified in the postcontrast studies and sagittal plane, showing a diffuse heterogeneous contrast enhancement. In our study, the kidneys showed homogeneous enhancement after contrast administration, and they were best identified in transverse and dorsal planes. The ureters could not be identified in chameleons [42], which is the same as in our study. The urinary bladder could not be observed during the study of [34] the green iguana, the common black and white tegus, and the bearded dragon; it is easy to identify in red-eared terrapins [37], whereas in chameleons, it was visualized in pre and postcontrast studies only in animals that presented with hyperattenuating urate salts in the lumen [42].

The only part of the reproductive organs identified in our study were ovarian follicles in one of the females, which were only on the right ovary. They appeared cranial to the ipsilateral kidney, which may be used as an anatomic landmark to find the ovaries. Valente et al. [29] pointed out the difficulty of identifying the reproductive organs in loggerhead sea turtles due to their small size and/or the lack of contrast with the surrounding tissues. However, in the study by Banzato et al. [45], the reproductive organs were identified only in two male bearded dragons, but they were not visualized in the other two bearded dragons or in the common black and white tegus and green iguanas included in their work. Di Ianni et al. [36] performed CT examinations of four reptile species (bearded dragons, Egyptian spiny-tailed lizards, blue-tongued skinks, and Sudan plated lizards) after contrast medium administration in the cloaca to improve the visualization of hemipenes in male individuals. They concluded that this technique is a feasible and sensitive tool for identifying the hemipenes and therefore for gender determination in these species of lizards. These authors suggest that identifying the hemipenes could be useful for gender determination in other nondimorphic lizards, especially in those species in which the use of ultrasounds in the coelomic cavity is not feasible or is difficult. Such is the case for *Pseudopus apodus*, since the hardness of its scales avoids the transmission of ultrasound waves (based on the author’s observations). Gumpenberger [47] reported that the testes are generally well differentiated in chelonians in precontrast CT images, appearing as homogeneous organs in a cranioventral position to the kidneys, and are clearly visible after intravenous contrast administration; differentiation is more difficult in snakes and lizards. This author considers CT as an excellent diagnostic imaging technique to identify follicles and eggs, thanks to a much larger gray scale and the lack of superimposition in the images compared to radiography. Sochorcová et al. [37] reported that ovarian follicles did not show postcontrast enhancement on CT and they were adequately seen in the precontrast images of red-eared terrapins. In the study by Melero et al. [42], the chameleons’ testes were identified in the majority of the male individuals and were located in the caudodorsal part of the coelom; they appeared as circular, bilateral, almost symmetrical, homogenous, soft tissue attenuating structures, which were mildly hypodense in comparison to the kidneys on precontrast images and showed mild diffuse contrast enhancement. In the same study, the ovaries were identified cranial to the kidneys in 40% of the female veiled chameleons, and they appeared as a cluster of multiple circular structures. The ovarian follicles, were observed with soft tissue attenuation in precontrast CT examinations, and mild diffuse enhancement of these structures could be observed in postcontrast studies. This is, at least partially, in accordance with our results, since we could also identify ovarian follicles only in postcontrast images. In the work of Melero et al. [42], when eggs were seen in chameleons, they appeared as ovoid structures with alternating hyper and hypoattenuating layers. A structural analysis of the female reptile reproductive system by micro-computed tomography (micro-CT) and optical coherence tomography (OCT) [48] has been recently published. Both are volumetric imaging techniques that offer exceptional quality images of the ovaries, infundibulum, glandular uterus, and non-glandular uterus, but from postmortem specimens, since they do not currently have clinical applications.

Although in our study the heart was identified in the two individuals in pre and postcontrast images, the differentiation between the atria and ventricle as well as the identification of the sinus venosus and great vessels, improved with intravenous contrast administration. Melero et al. [42] describe the CT image of the chameleons’ heart as a soft tissue attenuation structure with a round shape on the transverse plane and a more oval appearance on the sagittal plane that shows postcontrast enhancement. This is coincident with our results, although these authors mention that differentiation between the heart chambers was not possible even in their postcontrast series, and the latter differs from our findings. In the same work performed in chameleons [42], the cranial vena cava was visualized as a tubular longitudinal soft tissue attenuation structure localized on the most ventral aspect of the celomic cavity, and was slightly lateralized to the right side; the descending aorta was seen as a tubular structure, with a smaller diameter compared to the cranial vena cava, which was identified in the midline just ventral to the vertebral column. Both great vessels were more recognizable in the postcontrast series. In our study, we identified the sinus venosus, the two cranial cava veins, and the aortic arches near the heart, and the aorta in the midline just ventral to the spine.

Our study did not identify the smaller soft tissue organs, such as the spleen, pancreas, thyroid, oviducts, testes, and hemipenes, due to their size and adjacent positions to other organs with similar attenuation. The same reasons could be considered for the non-identification of the ureters, urinary bladder, and some major vessels, such as the pulmonary trunk and pulmonary arteries, that were identified in the anatomic dissection. It has been reported that the spleen can be identified in some reptiles like loggerhead sea turtles [29], green iguanas, and black and white tegus, but not in bearded dragons [34] or chameleons [42]. The pancreas can be identified in green iguanas [34], but it is difficult to distinguish in loggerhead sea turtles [29] and is not differentiated in black and white tegus, bearded dragons [34], or chameleons [42].

The use of microCT is quite extended for studying skeletal structures of reptile specimens, but it cannot be used for live animals. The skeletal anatomy of *Pseudopus apodus* has been thoroughly described using microCT [5,6,7,8,9,10,11,12,13], with a very high image resolution compared with that offered by multi-detector CT. Thus, although this work does not include a description of the axial or the appendicular skeletal structures of these individuals, as the main goal is the study of the coelomic cavity, nevertheless, in our study, multi-detector CT was able to provide high resolution of the hyoid bone.

## 5. Conclusions

This study provides a detailed description of anatomical dissection and multi-detector CT features of the non-skeletal structures of *Pseudopus apodus*. The vomeronasal organ with the choanae, the tongue, the glottis, the hyoid bone, the esophagus, the stomach, the small and large intestines, the cloaca, the liver, the gallbladder, the kidneys, the ovarian follicles, the trachea, the bronchial bifurcation, the lungs, the heart, the aortic arches, the aorta, the sinus venosus, and the cranial cava veins were identified in both the anatomic dissection and CT images. However, the thyroid, the pancreas, the spleen, the ureters, the urinary bladder, the oviducts, the testes, the hemipenes, the pulmonary trunk, and the pulmonary arteries were seen in the anatomical dissection but not in the CT studies. Our results demonstrate, for the first time, that multi-detector CT scanning is a useful tool for examining the numerous non-skeletal structures of *Pseudopus apodus*. In summary, our contribution to the knowledge of this animal, scarcely studied so far, is therefore important, and implies a significant advancement for different sciences, such as biology, anatomy, physiology, and pathology.

## Data Availability

The authors confirm that the data supporting the findings of this study are available within the article.

## Abbreviations

a	atria
aa	aortic arches
Ag	adrenal glands
ao	dorsal aorta
B	bladder
C	cloaca
cv	cranial cava vein
cvc	caudal cava vein
E	esophagus
el	eyelid
ey	eye
G	gallbladder
gt	glottis
Hp	hemipenis
hy	hyoid bone
li	large intestine
lg	lateral groove
lL	left lung
Kd	kidney
Lv	liver
m	mouth
L	lungs
ns	nostril
Od	ovarian duct
oc	otic cleft
oo	oocytes
O	ovary
P	pancreas
pe	parietal eye
pm	pterygoideus muscles
rL	right lung
S	stomach
Si	small intestine
Sp	spleen
sv	sinus venosus
t	teeth
tes	testicles
Th	thyroid gland
to	tongue
tr	trachea
v	ventricle
vn	opening of the vomeronasal organ and choanae
vs	venous sinus
